# Making Sense of the Relationship Between Ultra-Processed Foods, Obesity, and Other Chronic Diseases

**DOI:** 10.3390/nu16234039

**Published:** 2024-11-26

**Authors:** Norman J. Temple

**Affiliations:** Centre for Science, Athabasca University, Athabasca, AB T9S 3A3, Canada; normant@athabascau.ca; Tel.: +44-757-609-5352

**Keywords:** obesity, ultra-processed foods, processed foods, chronic diseases of lifestyle, hyperpalatable, energy density of food

## Abstract

Ultra-processed foods (UPFs) is a food category within the NOVA system. The key feature of UPFs are foods that have been highly processed and contain various additives, especially those that are industrially produced. It is claimed that UPFs are inherently unhealthy. The classification system is highly controversial. This paper critically evaluates the evidence. In stark contrast to conventional systems for food classification, the NOVA system disregards the nutritional values of foods. As a result, many foods generally considered to be healthy are included as UPFs, whereas many unhealthy foods are excluded. Epidemiological studies, mainly prospective cohort studies, have consistently reported an association between the intake of UPFs and risk of obesity, cardiovascular disease (CVD), type 2 diabetes, common mental disorders (especially depression), and all-cause mortality. A similar association has been reported for cancer and hypertension, but the supporting evidence is weaker. The most plausible explanation for this is that the associations are largely due to a limited number of unhealthy foods, such as processed meat and sugar-sweetened beverages. Studies of the relationship between UPFs and obesity present a different picture. There is much evidence that suggests that UPFs play a major causal role in obesity. The high contents of fat, sugar, carbohydrates, and sodium commonly present in UPFs makes these foods hyperpalatable. In addition, UPFs typically have a high energy density. As a result of these two features of UPFs, most people consume an excessive energy intake when presented with UPFs. Because UPFs include a wide range of foods, many of which are healthy, it is likely that while many UPFs are obesogenic, many others are not.

## 1. Introduction

An enormous body of research has emerged in recent decades that demonstrates that diet has a major impact on disease risk. In particular, the diet commonly eaten in the Western world (the “Western diet”) is closely associated with the risk of chronic diseases of lifestyle (CDL). Conversely, a healthy diet lowers the risk of these diseases. Based on these findings, many sets of recommendations have been made, with the goal of helping to prevent CDL [1,2,3,4].

The NOVA system represents a radically different approach to the classification of foods than nearly all other systems. In this system, foods are classified based on the degree of processing involved in their production. Within that system, one group of foods is classified as ultra-processed foods (UPFs). The system is based on the assumption that all UPFs are unhealthy. Accordingly, dietary recommendations based on the NOVA system recommend the reduction or elimination of UPFs. The NOVA system has steadily gained significant support from many nutrition experts. For example, Lawrence [5] stated, “UPF is a fit-for-purpose concept for guiding nutrition policy activities to tackle unhealthy and unsustainable diets”. Brazil has incorporated recommendations into their dietary advice for the general population that the intake of UPFs should be reduced [6].

This viewpoint has received much criticism in recent years. A debate is now underway in the pages of nutrition journals, with one side attacking the scientific rationale for classifying a wide variety of foods as UPFs [7,8]. This paper critically evaluates the evidence.

## 2. The NOVA System

The NOVA system was developed in Brazil by Monteiro and colleagues [9]. The system was later modified [10,11]. NOVA classifies foods into one of four food groups based on the extent and purpose of the processing they have undergone. The four food groups are as follows:

1. Unprocessed or minimally processed foods. These include fresh, dry, or frozen fruits or vegetables, grains, legumes, meat, fish, and milk.
2. Processed culinary ingredients, including sugar, oils, fats, salt, and other constituents extracted from foods and used in kitchens to make culinary preparations.
3. Processed foods. These include such foods as canned fish and vegetables, simple breads, and cheeses, which are manufactured by only adding salt, sugar, oil, or other processed culinary ingredients to unprocessed or minimally processed foods.
4. Ultra-processed foods (UPFs). These are foods that are made from mixtures of ingredients that are the products of industrial processes. UPFs have products extracted from actual foods combined with various additives.

In the original system, which was proposed in 2010, foods in group 3 were classed as UPFs and were therefore included together with all other UPFs [9]. By 2018, that single food group was divided into groups 3 and 4 [10,11]. The obvious explanation for this is that the researchers who developed the NOVA system realized that their definition of UPFs was seriously flawed. The evolution of the definition of UPFs was discussed by Gibney [12].

This classification system is altogether different from all conventional systems; it disregards the nutrient composition of foods. With other systems, the aim is to persuade people to eat a diet that consists mainly of foods that are strongly associated with a reduced risk of CDL while also providing appropriate amounts of all macronutrients and micronutrients. This approach is the guiding principle in the Dietary Guidelines for Americans [13]. We also see this with the design of food guides; recent versions used in different countries place a strong emphasis on the consumption of a diet rich in fruits, vegetables, and whole grains.

Front-of-package food labels use the same strategy. For example, Nutri-Score is a front-of-package food label that was developed in France [14,15]. Foods are placed in one of five categories based on their content, which includes a range of components. Foods are considered less healthy as the quantities of saturated fat, sugar, sodium, and energy increase but healthier with higher contents of protein, dietary fiber, fruits, vegetables, pulses, nuts, and healthy oils, such as olive and canola [16]. This is based on solid supporting evidence [17]. Nutri-Score was first implemented in France in 2017 and is now used in several other countries, including Belgium, Switzerland, Germany, and the Netherlands.

A variation in the conventional system for making dietary recommendations that has emerged in recent years is to focus on dietary patterns [18]. The best-known example of this is the recommendation to follow a Mediterranean diet [19]. These various sets of dietary recommendations share the common feature of being based on foods or dietary patterns where there is strong evidence that the recommendations, if followed, will lead to a reduced risk of CDL.

Forde [7] and Visioli et al. [8] made detailed critiques of the numerous flaws in the NOVA system. One anomaly in the system that should be highlighted is that foods are categorized in ways that ignore their likely impact on health. For example, many foods contain significant amounts of whole grains and are therefore likely to be healthy, but such foods can still be included in UPFs if the grain has been processed and they also contain several additives. This can be the case with some brands of bread. Conversely, red meat is often eaten as a steak and is therefore an unprocessed or minimally processed food. However, intake of red meat is associated with an increased risk of cardiovascular mortality [20] and of several types of cancer [21]. Similarly, many foods with added salt can be classified as a “processed food”, but if the food also contains several additives, it will probably be classified as a “UPF”. This ignores the fact that salt is arguably the most toxic of all food additives. The same inconsistency is seen with sugar. It is another substance added to many foods and is strongly linked to several types of CDL. In some cases, food with added sugar is classed as a “processed food” (e.g., coffee with sugar), but in other cases, it is a UPF (e.g., cola drinks).

An American study demonstrated that it is quite possible to design a diet based mainly on UPFs but that is relatively healthy [22]. In this study, a diet was designed based on the recommendations stated in the Dietary Guidelines for Americans [13]. It comprised foods commonly eaten in the USA. The large majority of food energy (91%) came from UPFs. While the diet had an excess of salt and a poor content of whole grains, it had adequate contents of all micronutrients, except vitamins D and E.

## 3. The Relationship Between Ultra-Processed Foods and Disease

Many epidemiological studies, mainly prospective cohort studies, have been carried out, where researchers investigated the possible association between the intake of UPFs and risk of CDL. The strongest and most consistent associations were reported for obesity, cardiovascular disease (CVD), type 2 diabetes, common mental disorders (especially depression), and all-cause mortality [23,24,25]. The evidence is weaker for cancer and hypertension. One notable feature of the findings is their lack of consistency. One factor that may help explain this is the quantity of UPFs consumed in the study population. This amount is at least twice as high in Northern Europe than in Southern Europe [26]. A major factor that is probably responsible for the inconsistencies between studies is the variation in which foods are classified as being UPFs.

In the above studies, UPFs were treated as a single food group, irrespective of the main types of foods included with the UPFs. However, other studies investigated the relationship between different types of UPFs and the risk of CDL. As pointed out earlier, UPF is an umbrella term that includes many unhealthy foods, as well as many healthy foods. It is therefore unsurprising that the relationship between UPFs and disease risk shows major differences between different types of UPFs. This was shown in the following cohort studies.

In the combined results from two cohort studies carried out in the USA, the association between UPFs and all-cause mortality was largely accounted for by ready-to-eat products based on meat (e.g., processed meat), poultry, and seafood, sugar-sweetened and artificially sweetened beverages, dairy-based desserts, and ultra-processed breakfast cereals (excluding whole grain products) [27]. A cohort study was carried out on renal transplant patients in the Netherlands [28]. The association between the intake of UPFs and all-cause mortality was entirely accounted for by sugar-sweetened beverages, processed meat, and desserts. A cohort study carried out in Europe studied the relationship between the intake of UPFs and the risk of a person developing both cancer and CVD [26]. A clear association was seen for sugar-sweetened and artificially sweetened beverages and for animal-based products but not for UPFs, such as breads and cereals, sweets and desserts, and plant-based alternatives.

Unfortunately, the weight of evidence is insufficient for definitive conclusions. However, the types of UPFs that seem to be most closely associated with an increased risk of CDL are processed meat and sugar-sweetened and artificially sweetened beverages. The findings on desserts are not consistent, possibly due to the different types of desserts commonly eaten in different countries. By contrast, UPFs with high contents of whole grains, fruits, or vegetables seem unrelated to the risk of CDL.

## 4. Ultra-Processed Foods and Obesity

The evidence linking UPFs with CDL is considerably stronger in the area of excess weight gain and obesity than appears to be the case with other types of CDL. The evidence that UPFs as a whole are a major factor in the causation of obesity was presented in a previous paper [29]. Additional evidence is presented here.

An epidemic of obesity emerged in the USA around 1976–80 [30]. It then steadily spread across the Western world. By the 1990s, the epidemic was clearly emerging in less affluent countries [31]. There has been a great deal of debate as to the cause of this epidemic. The growth in the intake of UPFs provides the best explanation [29]. In brief, there is solid evidence that UPF plays a causal role in obesity.

In order to more fully understand how some types of food tend to lead to obesity, it is essential to better understand the etiology of excessive food intake. One essential feature of eating is that people have very limited ability to control how much they eat. This was argued by Cohen and Farley [32], who concluded that “…eating [is] an automatic behavior, as opposed to one that humans can self-regulate”. The compositions of many types of UPFs bypass the appetite control system and induce an excessive energy intake. Gearhardt and Schulte [33] argued that UPFs act in a similar way to drugs in being addictive. UPFs that are most likely to trigger this addictive behavior are foods that have high contents of both fat and carbohydrates.

Dicken et al. [34] made a similar argument. In their view, the key feature of UPFs, with respect to excessive food intake, is their high content of fat, sugar, carbohydrates, and sodium. This composition results in the majority of UPFs being characterized as hyperpalatable. They demonstrated this based on an analysis of foods eaten in the UK. They divided foods according to the NOVA system and then calculated the proportion of foods in each group that met their definition of being hyperpalatable. Of those foods classified as being minimally processed, 13.4% were hyperpalatable, but this increased to 49.8% for processed food and 68.0% for UPFs.

Another key feature of UPFs that results in an excessive intake of food energy is a high energy density. In the above study of foods eaten in the UK, the researchers concluded that the median energy contents of minimally processed food, processed food, and UPFs are 94, 178, and 243 kcal/100 g, respectively [34]. The importance of this was demonstrated in a study by Klos et al. [35]. They conducted a systematic review and meta-analysis of 38 randomized controlled trials. They compared meals with higher and lower median energy densities (150 vs. 110 kcal/100 g). The higher energy density led to a higher energy intake of 223 kcal/day. This increased amount of food energy, if repeated at a single meal each day, was many times greater than that needed to account for the obesity epidemic.

That UPFs do indeed lead to an increase in energy intake was demonstrated in a randomized controlled trial [36]. Groups of subjects were fed either UPFs or a diet based on minimally processed foods. The subjects were free to eat as much food as they wished. Over the course of 2 weeks, subjects eating the UPFs diet consumed 508 kcal/day more food energy. This led to an average weight gain of 0.9 kg. This result of eating UPFs is not an accident. Food technologists have applied great skill to the design and manufacture of UPFs in order to make them as appealing and as enjoyable to eat as possible.

The evidence presented above demonstrates that UPFs, in general, are both hyperpalatable and have a high energy density in comparison with minimally processed foods. Foods classified as “processed”, according to the NOVA scheme, are in between but are closer to UPFs than to minimally processed foods.

## 5. Discussion

### 5.1. Ultra-Processed Foods and Chronic Diseases

This examination of the NOVA system compels the conclusion that it is based on badly flawed science. All conventional systems that make recommendations regarding which foods should be eaten are based on whether a food is likely to enhance health and prevent disease, as well as on the nutrient composition of the food. The NOVA system, by contrast, classifies foods into food groups based on the extent and purpose of the processing they have undergone. The key feature of the system is the claim that foods classified as UPFs (NOVA 4) are inherently unhealthy.

Multiple cohort studies have reported that the intake of UPFs is associated with an increased risk of several types of CDL. The strongest and most consistent findings have been seen for obesity, cardiovascular disease, type 2 diabetes, common mental disorders (especially depression), and all-cause mortality. The evidence is weaker for cancer and hypertension. It must be borne in mind that UPFs have much overlap with unhealthy foods. Accordingly, the association between UPFs and CDL does not imply that UPFs, as a broad food group, are causally related to the risk of CDL. Rather, UPFs may be acting merely as a crude indicator for the consumption of unhealthy foods. This does indeed appear to be the case. In the handful of cohort studies where the investigators reported on the relationship between different types of UPFs and the risk of CDL, the types of UPFs that seem to be most closely associated with an increased risk of CDL are processed meat, sugar-sweetened and artificially sweetened beverages, and possibly desserts. By contrast, there is no evidence that UPFs with high contents of whole grains, fruits, or vegetables are related to the risk of CDL.

### 5.2. Ultra-Processed Foods and Obesity

An impressive body of evidence has accumulated in recent years that strongly suggests that UPFs play a major causal role in obesity. Much of the evidence was presented in a previous paper [29]. The additional evidence presented here adds weight to the hypothesis. The high contents of fat, sugar, carbohydrates, and sodium commonly present in UPFs make these foods hyperpalatable. In addition, UPFs typically have a high energy density. These two features of UPFs probably act synergistically, with the result being that when given UPFs, most people consume an excessive energy intake. This behavior can be viewed as a form of hedonism, which the Cambridge Dictionary defines as “living and behaving in ways that mean you have as much pleasure as possible”. Such foods can be called hedonic (from H [hyperpalatable] ED [energy dense] onism).

However, it must again be stressed that UPFs include a wide range of foods, many of which are quite healthy. It is therefore likely that while many UPFs are obesogenic, many others are not. For example, some brands of bread can be categorized as UPFs but are relatively high in fiber and have a relatively low energy density. Such foods are unlikely to be either hedonic or obesogenic.

Table 1 presents a comparison between Nutri-Score (a front-of-package food label) and UPFs. This illustrates the key differences between the conventional approach to the evaluation of foods and the NOVA system. While Nutri-Score focuses on the risk of CDL and the nutritional value of food, the NOVA system focuses on the degree to which a food has been processed. Overall, the conventional approach to the evaluation of foods is clearly superior to the NOVA system. However, the NOVA system may have the advantage of highlighting the high probability that most UPFs are obesogenic.

### 5.3. Priority Areas for Research

The research findings discussed in this paper point to several areas where more research is needed. The relationship between UPFs and various types of CDL still has many gaps in the evidence. Further clarification is needed regarding the association between the intake of UPFs and different types of CDL. Of particular importance, more research is needed in order to determine which types of UPFs are most closely linked to the different types of CDLs.

These areas where more research is needed are especially relevant to the relationship between UPFs, energy intake, weight control, and obesity. The following are some of the key questions that require further investigation:

- Reference was made to a randomized controlled trial where subjects ate more food energy and gained weight when given a diet based on UPFs, compared with one based on minimally processed foods [36]. However, the duration was only 2 weeks. What will be the outcome with a longer duration, say 6 to 12 months? How will the outcomes be affected by changes in other variables (such as age and weight of subjects and types of UPFs)?
- What types of UPFs (and other foods) are most likely to be hyperpalatable and result in an excessive intake of food energy?
- Going one step further, what foods are most likely to be obesogenic based on being hyperpalatable, having a high energy density, and other characteristics (such as a soft texture and a low fiber content)?

### 5.4. Ultra-Processed Foods and Nutrition Advice

The evidence examined here has important implications for the nutrition advice that is delivered to the general population. It must be stressed that there are serious weaknesses in the claim that UPFs are a distinct type of food and are inherently unhealthy. For that reason, the inclusion of UPFs in food guides, front-of-package food labels, and similar documents adds little value and would therefore add more noise than signal. Let us now suppose that UPFs do indeed play an important role in the causation of obesity. This does not change the previous conclusion. A person who follows a typical food guide would eat a healthy diet and almost certainly cut down on his or her intake of obesogenic types of UPFs. Such a diet would be expected to greatly reduce the risk of obesity. However, as in many aspects of providing nutrition advice to the public, more research is a high priority. We must be open to the possibility that future research will reveal that specific advice to avoid UPFs, especially hedonic foods, will need to be included in nutrition advice for the population.

When it comes to formulating nutrition advice for health professionals, a different approach may be warranted. This is illustrated by a detailed statement published by the American Heart Association in 2021 that provides dietary guidance for promoting cardiovascular health and preventing CVD [37]. It was authored by leading American experts in the area of diet and health. The document presents ten sets of recommendations. Of these, nine are conventional sets of dietary recommendations, but one states the following: “Choose minimally processed foods instead of ultra-processed foods”. This document achieves an eminently sensible approach to dietary recommendations for health professionals.

## 6. Conclusions

Conventional systems for food classification are based on the nutritional values of foods and whether a food is strongly associated (positively or negatively) with the risk of CDL. By contrast, the key feature of UPFs is that they are highly processed and contain various additives, especially those that are industrially produced. Many UPFs are unhealthy, and others are reasonably healthy, while some unhealthy foods are not classified as UPFs. The case has been presented here that the NOVA system (including classifying many foods as UPF) is not consistent with the evidence from nutrition science. However, many cohort studies have reported an association between the intake of UPFs and risk of obesity, CVD, type 2 diabetes, common mental disorders (especially depression), cancer, hypertension, and all-cause mortality. These associations are best explained as being largely due to a limited number of unhealthy foods, such as processed meat and sugar-sweetened beverages. The relationship between UPFs and obesity presents a different picture. Here, UPFs do appear to play a major causal role. This is because many UPFs (but not all) are hyperpalatable and have a high energy density. As a result, most people consume an excessive energy intake when given UPFs.

## Figures and Tables

**Table 1:** Comparison between Nutri-Score and ultra-processed foods (UPF).

	Nutri-Score	UPF
Criteria for inclusion	Characteristics of food that are strongly associated (positively or negatively) with the risk of CDL (e.g., quantity of sugar and fiber).	Product of industrial processes. Ingredients have been extracted from actual foods and combined with additives.
Reliability as a predictor of whether the food increases or decreases the risk of CDL.	High	Moderate
Reliability as a predictor of weight gain and obesity	Moderate (but uncertain)	Possibly high (but uncertain)
Disadvantages	Does not consider whole grains, red meat, or fish	Inaccurate indicator of whether a food is healthy or unhealthy

The information in the table compares Nutri-Score (a front-of-package food label) with UPFs (one of the food groups within the NOVA system). Nutri-Score is an example of the conventional approach to the evaluation of foods. CDL: chronic diseases of lifestyle.
